# Three-dimensional assessment of finger individuation reveals finger- and joint-specific selective motor control deficits in pediatric cerebral palsy

**DOI:** 10.21203/rs.3.rs-6896151/v1

**Published:** 2025-06-25

**Authors:** Owais A. Khan, Divya Rai, Brooklyn Crabtree, De’Yana Hines, Gavin Colquitt, Christopher M. Modlesky, Jing Xu

**Affiliations:** Department of Kinesiology, University of Georgia, Athens, GA, 30602, USA.; Department of Kinesiology, University of Georgia, Athens, GA, 30602, USA.; Department of Kinesiology, University of Georgia, Athens, GA, 30602, USA.; Department of Kinesiology, University of Georgia, Athens, GA, 30602, USA.; Blue Cross NC Institute for Health & Human Services, Appalachian State University, USA.; Department of Kinesiology, University of Georgia, Athens, GA, 30602, USA.; Department of Kinesiology, University of Georgia, Athens, GA, 30602, USA.

**Keywords:** cerebral palsy, selective voluntary motor control, fine motor, hand function, finger individuation, dexterity, perinatal stroke, outcome measures, instrumented assessments, force analysis

## Abstract

**Background:**

Children with cerebral palsy (CP) exhibit impaired selective motor control (SMC) that contributes to poor hand function, but current clinical assessments lack the sensitivity to detect finger- and joint-specific deficits to guide rehabilitation strategies. This study aimed to determine the internal consistency and validity of an objective, instrumented assessment of selective finger control (individuation) in children with CP, and to examine its relationship to clinical measures of upper limb function.

**Methods:**

A custom-designed device recorded 3-dimensional isometric forces concurrently from all five fingertips to compute a composite metric of finger SMC (Individuation Index) for each tested finger and force direction. Group differences in individuation ability were quantified using linear mixed-effect models. Relationships between individuation and clinical assessments were assessed with age-controlled partial Pearson correlation.

**Results:**

Twenty-eight children with CP and 18 typically developing control children were included. The non-preferred arm was tested in most children with CP (n = 16), with the preferred arm tested in controls and the remaining CP cohort. Individuation Indexes demonstrated excellent internal consistency across groups (all R≥0.97). Children with CP exhibited lower individuation than controls in both the preferred (Cohen’s d (d)=0.73) and non-preferred arms, with deficits in the non-preferred arm more pronounced during finger flexion (d=1.48), in the index finger (d=1.52), and in those exhibiting mirror movements (d=0.56). Exploratory analysis in children with CP tested bilaterally (n = 6) revealed finger-specific hand differences, with lower individuation in the non-preferred hand limited to the index finger (d=1.36). Clinical scores indicated generally worse manual ability in children with CP than controls (d range = 0.70-1.38). Individuation Indexes were not related to clinical scores in either hand in children with CP or in controls (all *p* > 0.05).

**Conclusions:**

This study provides a consistent, valid, and sensitive method to quantify finger SMC in children with CP, revealing finger-, force direction-, and hand-specific impairments that highlight aspects of dexterity not captured by clinical assessments. Quantifying finger individuation enables more precise characterization of hand dysfunction, advancing mechanistic understanding and targeted intervention design for children with CP.

## 1. Introduction

Cerebral palsy (CP), the most common motor disability in childhood (1, 2), is a descriptive term (3) for a group of postural-movement neurodevelopmental disorders resulting from non-progressive damage or malformation of the nervous system during early development, leading to significant activity limitations (4). Objective quantification of impairments is essential to clinical decision-making and intervention planning in CP, prompting efforts to standardize clinical assessments and outcome measures (5-7). Motor impairments in CP (8) are broadly categorized as positive signs of excess muscle activity (i.e., hypertonia, primarily spasticity) (9) and negative signs reflecting insufficient magnitude and/or control of muscle activity, including muscle weakness (10), dyscoordination, impaired postural control (11), and reduced selective motor control (SMC) (12). Negative signs contribute more substantially to activity limitations (13) and are more responsive to rehabilitation (14). However, they may share neurophysiological underpinnings with spasticity (15) and most clinical assessments lack the specificity and sensitivity needed to distinguish between positive and negative motor signs, and/or to detect subtle sub-clinical impairments that can impede intervention effectiveness in children with CP.

Among negative signs, reduced SMC has received increasing attention for its adverse impact on functional outcomes in children with CP (16, 17). The NIH Pediatric Motor Disorders Taskforce defines reduced SMC as “the impaired ability to isolate the activation of muscles in a selected pattern in response to demands of a voluntary posture or movement” (12), which manifests clinically as obligatory patterns of muscle co-contraction called synergies (16). This clinical presentation is limb-specific in CP (18), and shares similar features as spasticity and weakness, necessitating thoughtful, directed assessment to guide intervention planning. Current assessments of SMC range from clinical observational tools that visually identify involuntary joint movement during isolated tasks (19, 20) to instrumented methods that quantify unintentional muscle activity (21, 22) or joint torques during isometric contractions (23, 24). As walking ‘correctly’ without the obligatory synergistic mass patterns is a common rehabilitation goal for families and children with CP (25), greater emphasis has been placed on evaluating SMC in the lower extremities (26, 27), and its impact on walking ability (28, 29) and postoperative outcomes (30, 31). Though impairments of manual dexterity are well documented in CP (32-36), the role of SMC in mediating these deficits has only recently been recognized (37, 38), spurring interest in SMC-directed interventions to improve hand function (39). This limited focus in CP contrasts with research in adults post-stroke (40) that leverages finger individuation, the ability to isolate the intended finger(s) away from other fingers, to predict composite hand function and improve dexterity through targeted SMC interventions (41).

The increasing recognition of upper limb SMC impairment following early brain injury has led to the development of two novel clinical assessments for children with CP – the Test of Arm Selective Control (19) and the Selective Control of Upper Extremity Scale (42). Greater emphasis has also been placed on SMC components within widely used and easily administered clinical assessments, such as the Dissociated Movement section of the Quality of Upper Extremity Skills Test (43) and the Fugl-Meyer assessment (44). However, both CP-specific SMC assessments (19, 42) rely on subjective clinician ratings on an ordinal scale, which highlight general loss of function across larger proximal joints but lack the granularity to detect impairments at individual fingers and distal joint control that is critical for dexterous function. These tools typically collapse distinct components of finger control, such as force-direction and joint-specific force metrics, into a single composite score or rating (e.g., ‘normal’, ‘impaired’, or ‘absent’), which risks oversimplification of motor deficits and overlooks finger- and joint-specific variability in SMC previously documented in adults with stroke (45, 46). Additionally, the reliance on visual observation and inability to differentiate selective joint control from weakness reduces sensitivity to subtle changes in joint forces or muscle activation (21), and ordinal ratings further constrain responsiveness, which limits the utility of these tools to detect small but clinically meaningful changes, or track intervention-induced SMC improvements.

A prominent aspect of upper limb SMC is finger individuation (47, 48), with independent finger movements deemed essential for dexterous hand function. Isolated finger movements are primarily mediated by corticospinal tract (CST)-driven regulation of motor unit recruitment. This regulation occurs via direct monosynaptic connections to spinal motor neurons (corticomotoneuronal system), and through indirect modulation of spinal interneuronal networks (49-51). Damage to the CST during early development in CP (52, 53) disrupts these intricate control systems and contributes to upper limb dysfunction. Greater CST injury has been linked to more severe reaching deficits and impaired hand use in children with arterial perinatal stroke (54). Notably, isolated CST damage produces severe and permanent deficits in dexterity, but less fractionated functions like power grip appear relatively spared (55), suggesting the biological systems supporting independent finger movements are distinct from those underlying strength (46, 56). Recent studies using instrumented individuation assessments in CP (57, 58) reported heightened finger interdependence (enslaving) in young adults with bilateral CP (57) and in children with unilateral CP (58), highlighting the potential of quantitative methods to objectively capture subtle SMC deficits across the lifespan and clinical spectrum of CP. However, these studies lacked the high-resolution kinematic and kinetic evaluations across all movement directions needed to accurately characterize individuation impairment patterns. Further, to date, no studies have conducted 3-dimensional analyses of finger- and joint-specific forces in children with CP, and relationships of finger individuation metrics with hand function have not been described in this group.

To this end, the current study aimed to determine the internal consistency, sensitivity, and discriminative (known-groups) validity of a novel finger individuation assessment in children with CP using the Hand Articulation Neuro-training Device (HAND)—a novel tool that concurrently measures 3-dimensional isometric forces from all five fingertips during isolated finger force control (59). Here we use sensitivity to describe how precisely our individuation metrics can distinguish between individuals with different levels of finger SMC, rather than its ability to detect change over time (60). We also aimed to assess the effects of finger tested, force direction, the presence of mirror movements, age, and sex on individuation ability. Relationships between individuation and clinical assessments of upper limb function were quantified in children with CP and typically developing control children. We hypothesized that our finger-individuation protocol would demonstrate good-to-excellent reliability, high sensitivity, and robust discriminative (known-groups) validity, evidenced by children with CP displaying lower individuation ability than similar-aged controls, with group differences varying across finger tested and force direction. In line with prior studies in adults with stroke that reported weak (45) or no correlations between finger individuation and arm function (61), we expected limited associations between individuation and upper limb clinical scores in children with CP. We also included an exploratory analysis comparing finger individuation ability between the preferred and non-preferred hand in a subset of children with CP, and hypothesized lower individuation in the non-preferred hand.

## 2. Methods

### 2.1. Participants

Children with spastic CP aged 5–11 y recruited for a separate randomized controlled trial (NCT03484078) from the Children’s Healthcare of Atlanta, local schools, pediatric rehabilitation centers, and the Cerebral Palsy Foundation were asked to participate in this study. Similar-aged typically developing children were similarly recruited as controls (Con) using the same recruitment strategies and locations. Children with spastic CP who could walk independently, follow instructions, and complete experimental tasks were included. Exclusion criteria for children with CP included (1) a prior long-bone fracture in both limbs, (2) bisphosphonate medication, (3) orthopedic surgery within the previous 6 months, (4) abdominal baclofen pump usage, or (5) botulinum toxin treatment within the previous 12 months. Typically developing children with no history of neurologic or motor disorders, height and body mass between the 5th and 95th age- and sex-based percentiles were included as controls. Exclusion criteria for controls included (1) history of chronic medication use known to impact the musculoskeletal system, (2) a fracture in the lower extremities within the past year, or (3) sustained participation in high-level physical activity.

#### Functional classification and clinical assessments

Manual ability for children with CP was classified by the parents’ report using the Manual Ability Classification System (MACS) (62). Those at MACS level I could handle most objects easily and successfully, while those at MACS level II could handle most objects but with somewhat reduced quality and/or speed of achievement. Gross motor function was assessed by a trained physical therapist using the Gross Motor Function Classification System (GMFCS) (63). A GMFCS level I indicates independent ambulatory ability with some limitation in running or jumping, while a GMFCS level II indicates inability to run and jump with some limitation in walking long distances. The presence of spasticity, dystonia, or mixed tone (co-occurrence of spasticity with dystonia) was assessed using the Hypertonia Assessment Tool (64). Mirror movements were visually assessed as present or absent during repeated fist opening-closing of one hand in isolation while keeping the non-tested hand as still as possible (20).

### 2.2. Experimental Design and Procedures

#### Participant setup

**Figure 1:**
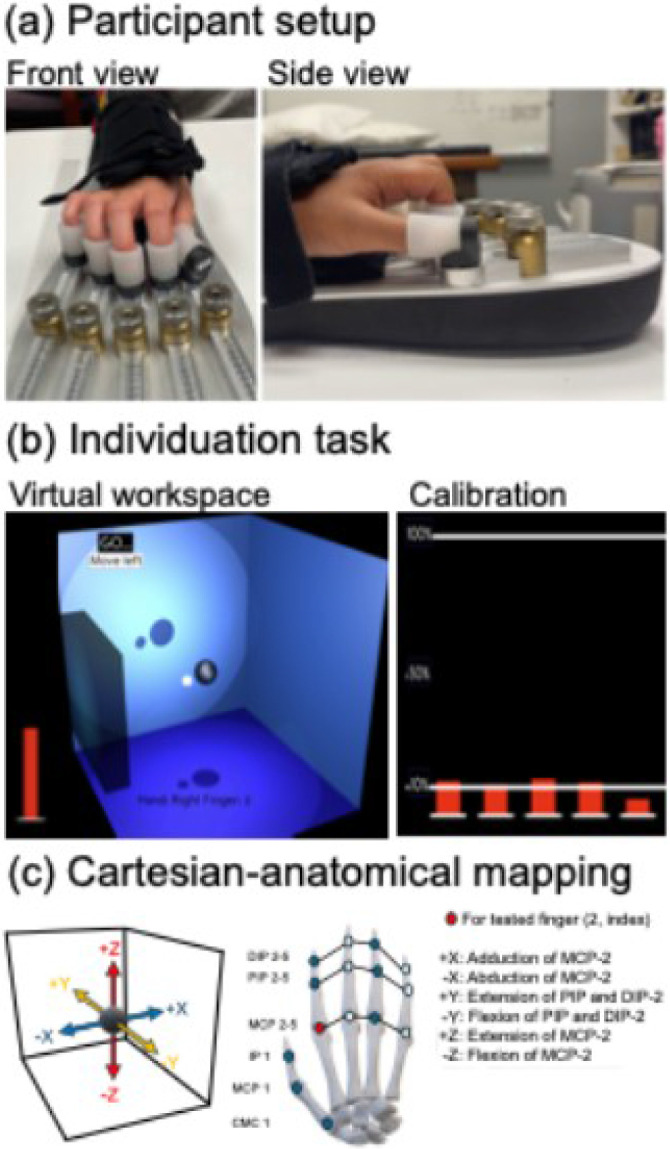
Experimental setup and the 3-dimensional finger individuation task. (**a**) Participant setup and positioning in the Hand Articulation Neuro-training Device (HAND). The tested hand was pronated, fitted with an adjustable brace, and secured to the HAND. Each fingertip was placed in a flexible silicone finger cup, and finger positions were adjusted by manipulating each sensor arm angle and length (top view) till a comfortable resting posture was obtained (side view). (**b**) Final resting position was calibrated as the hand posture that minimized overall fingertip forces to <1N (i.e., < 10% of the 10 N maximum, red bars on computer screen). This resting position was maintained across all trials. The tested hand, finger, and force direction were presented onscreen in a 3-dimensional virtual workspace alongside a movement cue signaling trial onset. Participants used one (instructed) finger to control the position of an onscreen ball (white sphere) within a virtual workspace (blue box). The task required repeatedly moving the ball from the central start position (gray sphere) towards a direction-specific target (gray wall) and back within the 15-second trial. Real-time performance feedback was provided with the target wall turning lighter shades of blue as the cursor moved closer. A red bar of variable height indicated the summed force from all uninstructed fingers, with participants instructed to minimize the bar height by restricting movement in these fingers. (**c**) Illustration of anatomical finger joint forces mapped to Cartesian coordinates in the virtual space. Each target direction corresponded to specific joint forces in the instructed finger. For example, the metacarpophalangeal (MCP) joint in the right-hand index finger corresponds to the x- and z-axes with adduction (+x), abduction (−x), extension (+z), and flexion (−z), while the proximal and distal interphalangeal joints (PIP and DIP, respectively) correspond to the y-axes with extension (+y) and flexion (−y).

Participants were comfortably seated with the trunk supported at a height-adjustable table. The tested forearm was positioned in a pronated posture with a soft brace that fastened to the HAND (59, 65) (Fig. 1a; also see Supplement 1). Each fingertip was fitted with a flexible silicone cup attached onto the device, and finger positions were individually adjusted to a comfortable resting posture that elicited minimum forces (< 1 N, Fig. 1b). This resting posture was maintained by fixing the knobs on the HAND and the mounting angle and distance of the finger cups from the adjustment knobs were recorded. All children with CP attempted the protocol with the non-preferred hand. The preferred hand was tested instead in those who could not complete the task with the non-preferred hand. As previous literature observed no differences in finger individuation between hands in typically developing children (58), the preferred hand was tested for all controls.

#### Finger individuation task

The finger individuation task (Fig. 1b, see video in Supplement 2) is a simplified version of that previously described (65, 66). Briefly, participants were instructed to exert force with one fingertip (instructed finger) to control an onscreen cursor (white ball) in a virtual 3-dimensional space from a fixed starting location (central black sphere). Only instructed fingertip forces produced cursor movement. Participants were instructed to keep the other fingers as still as possible, with summed forces from the non-instructed fingers reflected by the height of a vertical red bar that participants were instructed to minimize throughout the trial. Force direction was specified by the appearance of a virtual wall. Participants were asked to move the cursor from the start position (centrally located sphere), hit the target wall, and return to the sphere repeatedly within the 15 second trial duration. Real-time performance feedback was provided by increase in brightness of the virtual wall as the cursor approached the target. Cursor movement in the virtual Cartesian space were mapped to the joint space (65) (Fig. 1c). For example, index finger force change along the x-axes corresponded to metacarpophalangeal joint adduction (+x) and abduction (−x), and that along the z-axes corresponded to metacarpophalangeal extension (+z) and flexion (−z), while force changes along the y-axes corresponded to proximal and distal interphalangeal joint extension (+y) and flexion (−y). One trial was conducted for each of the six directions for each tested finger (thumb, index, and ring finger, in pseudorandomized order), resulting in 18 trials per session. Breaks were provided between trials as needed, and verbal encouragement and positive reinforcement was provided to optimize participant engagement.

### 2.3. Force data processing

**Figure 2:**
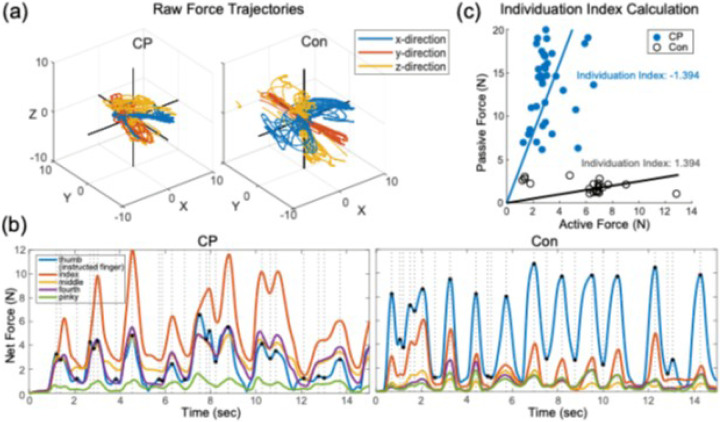
Computation of the finger Individuation Index. (**a**) Sample raw force trajectories from a child with cerebral palsy (CP) and a typically developing control child (Con) pooled across force directions during the individuation task. (**b**) Identification of force peaks and troughs in the instructed fingers and calculation of mean deviation from baseline force (*meanDevP*) in the non-instructed fingers across the 15-second trial duration. The net force trajectories of the instructed finger (thumb, blue line) are visualized alongside the net force trajectories of the uninstructed fingers (other colored lines). Peaks and troughs in instructed finger forces were identified (black dots, dotted lines signifying time-points), and uninstructed finger forces during intervals between successive peaks was calculated as the root mean square (RMS) and summed across all uninstructed fingers (*meanDevP*). (**c**) Illustration of Individuation Index derivation. A robust linear regression function was estimated for instructed finger forces against the *meanDevP* from the non-instructed finger forces, with the Individuation Index computed as the −log(slope) of the robust regression line.

Data were processed using custom MATLAB (MathWorks, Natick, MA) programs. Force data were smoothed using a low-pass filter (2nd order, cutoff frequency = 5 Hz), converted to Newtons, and normalized using the mean of the first 100 data samples (baseline). Raw force trajectories were processed for each trial. As participants completed multiple movements towards the target position (Fig. 2a), often with varying magnitude of forces, instructed finger peak and trough forces (magnitude, timing) were identified for each force trajectory. Finger individuation ability was assessed for each instructed direction in 3-dimensions using an Individuation Index, as detailed in prior work by our group (46, 65). Briefly, forces at the instructed finger were identified at each time-point when its net force trajectory (summed over the x-, y-, and z-directions) reached a peak or a trough during each trial (Fig. 2b). Forces from the uninstructed fingers were computed as the mean deviation from baseline forces (*meanDevP*, Eq. 1) during intervals spanning consecutive peaks in the active force trajectory.


(Equation 1) $$meanDevP=\frac{1}{T_{k}}{\sum \;}_{t_{k}=init}^{T_{k}}\sqrt{{\sum \;}_{j=uninstructed}{\left(F_{t_{k}j}-F_{jbaseline}\right)}^{2}}$$

Specifically, the root mean square of forces from all uninstructed fingers were computed at all time points from the preceding peak in the instructed finger active force $\left(t_{k}\right)$ to the current trough in the same active force trajectory $\left(T_{k}\right)$. The *meanDevP* was calculated for all intervals spanning each pair of successive peaks and troughs in the instructed finger active net force trajectory, for each trial. The Individuation Index was calculated using the slope of a robust regression function of *meanDevP* across all uninstructed fingers against active force at the instructed finger (Fig. 2c). The Individuation Index was then normalized using log transformation and multiplied by −1 [Individuation Index= -log(slope)], so that more negative values (higher slopes) represent lower individuation ability. A single Individuation Index was calculated for each force direction (x-, y-, and z-directions) by each instructed finger.

Internal consistency of the computed Individuation Index was assessed for each group using the split-half reliability method (75). For each trial, peaks and trough data points were randomly split to two halves for 10 times, and an Individuation Index was computed from each half. Correlations between the two halves were then computed. As there is increased variability when splitting data into halves, the formula $R=\frac{2r_{p}}{r_{p}+1}$ was used, where $r_{p}$ is the mean of correlations across all 10 splits. Split-half reliability metrics indicated excellent internal consistency for Individuation Indexes in the non-preferred (R=0.97) and preferred (R=0.98) hands in CP, and in the control group (R=0.98), in line with our previous work in adults with stroke (46, 65).

### 2.5. Clinical assessments of upper limb function

Standardized clinical assessments were used to evaluate manual ability and dexterity. The Purdue Pegboard Test (PPT) (67) measures unimanual dexterity through the number of standardized pegs a participant can sequentially place into a pegboard in 30 seconds (68). A higher number of pegs placed indicates better fine manual ability (69). The Box and Blocks Test (BBT) (70) measures gross unimanual ability through the number of 1-inch blocks moved from one side of a partitioned box to the other in 60 seconds (71), with a higher number of blocks moved indicating better gross manual ability. The Functional Dexterity Test (FDT) (72) measures in-hand manipulation as a component of dexterous speed, and is computed as the number of pegs turned per second when a participant attempts to flip 16 pegs in a specified pattern (73). Slower speeds indicates lower manual dexterity (74).

### 2.5. Statistical Analyses

Group differences in physical characteristics were assessed using Independent t-tests for data that were normally distributed, and with Mann-Whitney *U* tests for non-normally distributed data. Linear mixed-effect model (LMM) analyses implemented in RStudio with the *lmerTest* package (76) were used to test differences in Individuation Indexes across conditions. Participant was included as a random factor to account for systematic variability introduced by subjects, while Group (CP vs Con), Finger (thumb, index, and ring), Force Direction (flexion, extension, ab/adduction), Mirror Movement (present, absent), Age, and Sex were included as fixed factors. In a separate exploratory, repeated-measures analysis restricted to participants with CP who completed testing with both hands, Hand (preferred, non-preferred) was included as a fixed factor to examine within-subject differences in individuation ability. Significant effects were followed by post-hoc comparisons using the *emmeans* package (77) with alpha set at 0.05 and correction for multiple comparisons done via the Benjamini-Hochberg method (78). Student’s t-tests were conducted to compare clinical scores across groups. Partial Pearson’s correlations (r) correcting for age were used to assess relationships between Individuation Indexes and log-normalized clinical scores in each arm in children with CP, and in the preferred arm in controls. Magnitude of effects were quantified using Cohen’s d (d), with cut-off values of 0.2, 0.5, and 0.8 indicating small, medium, and large effects, respectively (79).

## 3. Results

Twenty-eight children with CP ($n_{\mathrm{non}\text{-}\mathrm{preferred}\;\mathrm{arm}\;\mathrm{only}}=16$, $n_{\mathrm{preferred}\;\mathrm{arm}\;\mathrm{only}}=6$, $n_{\mathrm{both}\;\mathrm{arms}}=6$) and 18 typically developing control children (all preferred arm) participated in the study (Table 1). No group differences were observed for any physical characteristic (all *p* > 0.05).

### Individuation deficits in the non-preferred hand of CP vary by finger, force direction, and mirror movements

**Figure 3:**
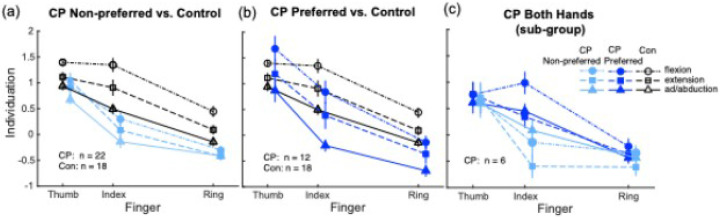
Finger Individuation Indexes across force direction (flexion, extension, ab-/adduction) and finger (thumb, index, ring) in each hand in children with cerebral palsy (CP) and in the preferred hand in typically developing control children (Con). **(a)** Comparison of individuation indexes between the non-preferred hand in children with CP versus controls. **(b)** Comparison of individuation indexes between the preferred hand in children with CP versus controls. **(c)** Comparison of individuation indexes between the preferred and non-preferred hands in a sub-group of children with CP who had both hands tested. Positive individuation indexes reflect greater individuation ability, while negative values indicate reduced individuation ability.

We used LMMs with Participant as a random effect and Group, Force Direction, Finger, Mirror Movements, Age, and Sex included as fixed effects to evaluate differences in finger individuation between the CP non-preferred hand and the control preferred hand (Fig. 3a). A Group × Force Direction interaction was observed after controlling for Finger (*p* < 0.001), with children with CP displaying lower Individuation Indexes than controls in all directions. Group differences were greatest in flexion (*d* = 1.48, *p* < 0.001) followed by extension (*d* = 1.09, *p* < 0.001) and ab-/adduction (*d* = 0.68, *p* = 0.001). Both groups exhibited higher Individuation Indexes in flexion compared to extension ($d_{CP}=0.30$, p=0.031; $d_{Con}=0.68$, p<0.001) and ab-/adduction ($d_{CP}=0.34$, p=0.006; $d_{Con}=1.13$, p<0.001). However, differences in individuation between extension and ab-/adduction were observed in controls ($d_{Con}=0.45$, p=0.001), but not in children with CP ($d_{CP}=0.04$, p=0.780).

After controlling for Force Direction, a Group × Finger interaction (*p* < 0.001) revealed lower Individuation Indexes in children with CP compared to controls across all tested fingers. Group differences were greatest for the index finger (*d* = 1.52, *p* < 0.001), followed by the ring finger (*d* = 0.90, *p* < 0.001) and thumb (*d* = 0.58, *p* = 0.003). Within both groups, individuation was higher in the thumb compared to the index ($d_{CP}=1.31$, p<0.001; $d_{Con}=0.37$, p=0.010) and ring finger ($d_{CP}=1.92$, $d_{Con}=1.67$; both p<0.001). Index finger individuation was also higher that the ring finger in each group ($d_{CP}=1.67$, $d_{Con}=1.29$; both p<0.001).

A main effect of Mirror Movements was also observed (*p* = 0.003): children with CP who exhibited mirror movements also demonstrated lower individuation ability than those without mirror movements (d=0.56, p=0.004), and lower individuation than controls (d=1.19, *p* < 0.001). No interactions were observed for Sex or Age (all *p* > 0.05). However, a main effect of Age (*p* = 0.016) suggested age-related improvements in finger individuation, with 12-year-olds displaying better individuation ability than 5-year-olds across the combined cohort (*d* = 0.56, *p* = 0.020).

### Individuation deficits in the preferred hand in CP are consistent across fingers and force directions

Separate LMMs with Participant as a random factor and fixed effects for Group, Force Direction, Finger, Mirror Movements, Age, and Sex were used to assess patterns of finger individuation in the preferred hand of children with CP and controls. Though no Group × Finger or Group × Force Direction interactions were observed (both *p* > 0.05; Fig. 3b), a LMM combining the 3 factors revealed significant main effects for each factor. Specifically, a main effect of Group (*p* = 0.001) was observed, with children with CP demonstrating lower overall finger individuation than controls (*d* = 0.73, *p* = 0.001). A main effect of Force Direction was also observed (*p* < 0.001), with individuation in the combined cohort higher during flexion compared to extension and ab-/adduction movements (*d* = 0.67 and 1.05 respectively, both *p* < 0.001), and higher during extension than ab-/adduction movements (*d* = 0.39, *p* = 0.001). Further, a main effect of Finger was also observed, with higher individuation in the thumb compared to the index and ring fingers in the combined cohort (*d* = 0.53 and 1.90, respectively; both *p* < 0.001). Individuation in the index finger was also higher compared to the ring finger (*d* = 1.90, *p* < 0.001).

While Group × Age and Group × Sex interactions were not observed (both *p* > 0.05), a significant main effect of Age (*p* = 0.003) revealed better finger individuation with increasing age in the combined cohort, with the oldest children at the age of 12 years displaying higher individuation indexes than the youngest participants at the age of 5 years (*d* = 0.66, *p* = 0.004). There was no effect of Mirror Movements on finger individuation (*p* = 0.212).

### Preliminary evidence suggests hand differences in individuation ability in CP are finger-specific

Exploratory within-subject analyses were conducted using separate LMMs in a small subset of participants with CP (n = 6) who completed bilateral testing (Fig. 3c). Models included Hand, Finger, and Force Direction as fixed effects, with Participant as a random factor. A Hand × Finger interaction was observed after controlling for Force Direction (*p* = 0.001). Individuation was higher in the preferred hand compared to the non-preferred arm for the index finger (*d* = 1.36, *p* < 0.001), while no differences were observed between hands for the thumb or ring finger (*d* = 0.15 and 0.22, respectively; both *p* > 0.05).

### Hand functional deficits observed on clinical assessments in CP are not linked to finger individuation ability

**Figure 4:**
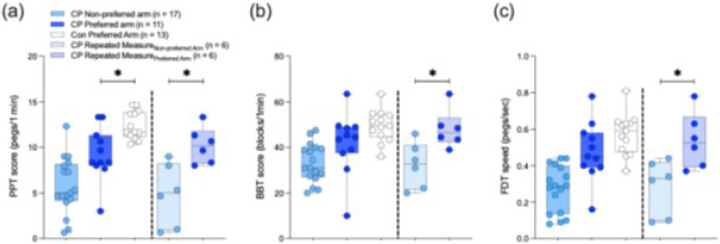
Upper limb clinical test scores and their relationship with finger individuation ability. Clinical assessment scores for (a) fine manual ability (Purdue Pegboard Test, PPT), (b) gross manual ability (Box and Blocks Test, BBT), and (c) in-hand manipulation (Functional Dexterity Test, FDT) in the preferred and non-preferred hand of children with cerebral palsy (CP) and typically developing control children (Con). A subset of children with CP (n = 6) completed clinical assessments with both hands, with within-group comparisons illustrated for each clinical assessment. Relationships of finger individuation indexes with log-normalized clinical scores for (d) fine manual ability (PPT), (e) gross manual ability (BBT), and (f) in-hand manipulation (FDT) in each hand in children with CP, and in the preferred hand in controls.

Among participants who completed the finger individuation task, 22 children with CP ($n_{\mathrm{Preferred}\;\mathrm{arm}}=11$, $n_{\mathrm{Non}-\mathrm{Preferred}\;\mathrm{arm}}=17$; each including $n_{\mathrm{Both}\;\mathrm{arms}}=6$) and 13 typically developing control children completed the three clinical tests (Fig. 4a-c; Table 2). Children with CP displayed impaired fine manual ability with the preferred arm, with fewer pegs inserted on the PPT compared to controls (*d* = 1.38, *p* = 0.003; Fig. 4a). While not statistically significant, similar trends were also observed for preferred arm gross manual ability, with generally fewer blocks moved on the BBT (*d* = 0.72, *p* = 0.092; Fig. 4b) in CP than controls, and similarly impaired in-hand manipulation, observed with slower speeds on the FDT (*d* = 0.70, *p* = 0.102; Fig. 4c) in CP than controls. Within-group analyses of the six children with CP who completed clinical tests with both arms revealed worse upper limb function in the non-preferred arm compared to the preferred arm (Fig. 4a-c), with fewer pegs inserted on the PPT (*d* = 1.98, *p* = 0.040; Fig. 4a), fewer blocks moved on the BBT (*d* = 1.70, *p* = 0.007; Fig. 4b), and slower in-hand peg manipulation on the FDT (*d* = 1.67, *p* = 0.036; Fig. 4c). Age-controlled partial Pearson correlational analyses (Table 2) revealed no significant associations between overall finger individuation ability and clinical assessment scores in either arm of children with CP, or in the preferred arm in controls (all *p* > 0.05).

## Discussion

This study investigated the reliability and sensitivity of an assessment tool for finger individuation using the HAND, a novel device that captures simultaneous 3-dimensional isometric force measurements from all fingertips. Our observations support its utility as a reliable, valid, and sensitive tool for assessing selective finger control in children with early brain injuries. Children with CP exhibited bilateral finger individuation impairments, with patterns of deficits appearing hand-specific. Generalized individuation deficits were observed in the preferred hand in CP which did not differ by finger tested, force direction, or the presence of mirror movements, with age-related improvements in individuation ability similar across groups. In contrast, the non-preferred hand in CP demonstrated widespread deficits that varied by finger and force direction, and were linked to the presence of mirror movements. The index and ring fingers demonstrated the greatest impairment in individuation ability, and flexion movements were most prominently impaired. Subgroup analyses comparing finger individuation across the two hands in children with CP revealed lower individuation specifically in the index finger of the more impaired hand, after controlling for force direction, reflecting findings from clinical assessments and reinforcing the discriminative capacity of our novel individuation paradigm. Contrary to the a priori hypothesis, finger individuation was not related to clinical test performance in either group; these observations suggest our individuation paradigm probes a unique aspect of finger control than current clinical assessments, which lack the sensitivity to detect compensatory strategies, or display a ceiling effect. These results extend prior work by offering objective and fine-grained metrics of upper limb SMC that highlights the HAND’s potential as a sensitive tool for assessment and individualized rehabilitation planning in CP.

### Finger- and movement-specific patterns of individuation vary by tested hand in CP

Variable patterns of individuation deficits were observed across fingers and force directions in the non-preferred hand in children with CP, with the index and ring fingers showing prominent deficits bilaterally compared to relatively preserved individuation in the thumb specifically within the preferred hand. These observations generally align with those reported in older adults with stroke (45, 61, 65), with some notable exceptions. Lang et. al. (45) similarly reported bilateral sparing of thumb individuation in adults with pure motor hemiplegia, though the index finger displayed only slight impairment compared to prominent deficits observed in our younger CP cohort. In contrast, individuation deficits were uniformly observed across all five fingers in patients with subcortical lacunar stroke exhibiting more impaired hand function (61), with higher likelihood of CST damage in this group posited to contribute to their greater individuation impairments. These discrepant findings may be explained by developmental differences in individuation (80) and enhanced neuroplasticity after early brain injury (81, 82). Additional contributors include methodological differences across studies, with the multidimensional HAND protocol assessing joint forces (i.e., kinetics) across 6 directions compared to the unidirectional flexion-extension kinematic protocol used in other studies (45, 61).

Individuation in the non-preferred hand in CP was more impaired during flexion than extension or ab-/adduction. We (65) previously reported individuation in the paretic hand of older adults with chronic stroke was least impaired during flexion movements. Neurophysiological differences between neurodevelopmental and adult-onset brain injury may underlie these discrepant findings. Adult-onset stroke is commonly associated with weakness and selective motor control impairments affecting the extensor muscles more than flexor muscles that are coupled with a bias toward co-contraction of these muscles in a stereotypical flexor synergy pattern (83, 84). The flexor synergy commonly described in adult-onset stroke (84) is hypothesized to be driven by compensatory recruitment of the rubrospinal (16) and/or cortico-reticulospinal pathways (85) due to the loss of CST input following neural injury (86). In contrast to direct CST inputs to distal extensors and hand intrinsics that support fractionated movements (49), the cortico-reticulospinal tracts supply multiple segments of the spinal cord (50), diffusely innervating multiple proximal arm muscles and distal flexor muscles. Given these neurobiological characteristics, compensation by extra-pyramidal motor systems such as the reticulospinal and rubrospinal tracts after CST damage may result in a stronger flexor synergy but poorer selective control in the flexion direction. However, flexor synergy is expressed minimally in the upper limb of children with CP (18), and differences in synergy patterns are dependent on the timing of early brain injury (23). Children with CP arising from post-natal lesions exhibited flexor synergies and minimal wrist-finger extensor activity, similar to the reticulospinal tract-driven patterns displayed by individuals with adult-onset stroke. Conversely, prenatal lesions commonly involve white matter damage (e.g., periventricular leukomalacia), with loss of contralateral corticospinal fibers compensated by preserved ipsilateral CST projections, with preserved finger and wrist extension capabilities (23). Spared ipsilateral CST connections following prenatal brain damage also underlie mirror movements (87) that commonly persist beyond early childhood and contribute to functional deficits in CP. These involuntary, obligatory movements presumably arise from preserved ipsilateral CST projections that strengthen in the absence of competitive pruning signals from the lesioned hemisphere following early brain injury (88, 89). Notably, we observed children with CP who displayed mirror movements had lower individuation in the more impaired hand compared to those who did not display mirror movements, further supporting our contention that patterns of individuation deficits are influenced by brain injury timing.

Further, Xu et al. (65) did not find differences in individuation between the non-paretic hand in individuals with stroke and the dominant hand of young neurotypical controls, contrasting with this study’s observations of significant differences between preferred hand individuation in children with CP compared to both controls, and to their non-preferred hand. However, these results are consistent with prior research in children with unilateral CP (58) indicating similar individuation deficits in the preferred hand compared to their non-preferred hand, and to controls. Interestingly, finger individuation metrics did not differ across hands in adults with spastic diplegic CP (57), though individuation was lower than neurotypical controls for each hand, with magnitude of group differences larger in the preferred $\left(\eta_{p}^{\;\;2}=0.62\right)$ than the non-preferred hand $\left(\eta_{p}^{\;\;2}=0.35\right)$. These contrasting observations underscore developmental differences in dexterous impairment across the CP lifespan (90), and highlight distinct recovery mechanisms in adult- versus childhood-onset brain injury due to enhanced developmental neuroplasticity (89) that warrants further investigation.

### Finger individuation may assess a different aspect of hand control than clinical assessments

The absence of significant associations between finger individuation and upper limb clinical assessments in typically developing children and CP suggests these assessments may capture fundamentally distinct aspects of motor function, supporting joint-level control versus multi-finger coordination during whole hand/arm movements. The clinical assessments used in this study demand multi-effector coordination (e.g., tripod pinch, precision grasp) and object manipulation, but do not interrogate the fine-grained, joint-level control quantified by the HAND. Alternatively, this dissociation may reflect ceiling effects of clinical assessments, however, the lack of associations in the more impaired arm in CP argues against this hypothesis. These findings are consistent with prior work in stroke (46), where individuation and strength were dissociated once strength reached moderate levels (~ 60%) of recovery. Collectively, these observations support finger individuation as a distinct and clinically meaningful construct in CP-related hand dysfunction.

### Strengths and Future directions

A key strength of this study is the use of a novel device capable of capturing finger- and direction-specific finger forces across 3-dimensions and over multiple movement planes, allowing the quantification of motor deficits likely undetected by conventional clinical evaluations. The HAND protocol demonstrated high sensitivity and robust reliability in detecting individuation impairments in children with CP. These findings build on strong biological evidence supporting the disproportionately greater involvement of distal joints following CST damage early in development (49). Despite evidence of disproportional involvement of distal joints (91, 92), current SMC assessments in CP (19, 42, 93) rarely probe joint- and movement-specific impairments in the hand, limiting their ability to guide targeted interventions. The HAND provides a promising approach to fill this gap. The importance of fractionated finger movement in daily tasks such as typing, using a keyboard, interaction with touchscreen digital devices or tools such as keypads makes the HAND a valuable assessment tool with sufficient sensitivity and reliability to interrogate this ability crucial for daily living among children with neurological damage.

This study opens several avenues for future research. Longitudinal studies are needed to characterize developmental trajectories of finger individuation in both typically developing children and those with CP. Co-occurring sensory-perceptual deficits and weakness may influence individuation outcomes (94), and merit integrated assessment in future investigations. In adults with stroke, individuation has been linked to strength recovery in a threshold-dependent manner (46), yet no comparable insight exists for children with early brain injuries. Examining strength and individuation and their interaction (95) may offer new insights on functional motor recovery in individuals with CP.

### Limitations

The modest sample size, though significantly larger than prior individuation-focused studies in pediatric (n = 4 (58)) and adult CP (n = 9 (57)), may limit the generalizability of our findings. However, the inclusion of a tightly controlled comparison group of typically developing children whose physical characteristics did not differ from age- and sex-based population norms in the current study increases confidence in the study findings. The absence of neurophysiological or neuroimaging data in the current study precludes insights into neural substrates of individuation abilities. Future work may consider incorporate neuroimaging and neurophysiological methods such as mobile functional neuroimaging via functional near-infrared spectroscopy (96), sensory-motor tractography, or transcranial magnetic stimulation-based assessment of CST integrity (97) to explore neural correlates of impaired finger individuation. While our cross-sectional study design provides valuable insights into motor control, longitudinal studies are needed to delineate developmental trajectories, establish stability, and confirm responsiveness of individuation metrics to change following intervention. Additionally, though all participants successfully completed the individuation task, attentional differences may have influenced performance in children with CP. While the protocol included audiovisual cues and performance feedback, attentional capacity was not formally assessed. However, most participants with CP also successfully completed the PPT, a task requiring sustained cognitive engagement (98), supporting our contention that motor control impairments, rather than attentional deficits, were the primary contributors to poor individuation in the CP cohort. Further, time restraints and concerns of fatigue restricted bilateral individuation evaluations to only 6 children with CP. Despite this small sample, preliminary sub-group analyses revealed hand differences in finger individuation, which aligns with prior research in CP (57) and reinforces the need to assess both hands even in children displaying unilateral affection (99). Finally, including children with more severe impairments (i.e., MACS levels III-V) and different CP subtypes (e.g., dyskinetic or ataxic CP) would enhance the clinical relevance of these findings.

## Conclusion

This study demonstrates that children with CP exhibit significant impairments in finger individuation that are finger- and force direction-specific, with these fine-grained metrics distinct from composite metrics provided by common clinical assessments. These findings support the HAND protocol as a valid, reliable, and sensitive method for quantifying nuanced finger individuation deficits across digits and directions in children with CP. Finger individuation assessments can provide critical insights into the intricate control mechanisms underlying dexterous function, and hold significant potential to inform the design of targeted rehabilitation strategies to improve hand use after developmental brain injury.

## Supplementary Material

This is a list of supplementary files associated with this preprint. Click to download.

• Supplement1KhanOACPFingerIndiv.docx

• Supplement2KhanOASPFingerIndividVideo.mp4

## Figures and Tables

**Table 1:** Participant characteristics of children with cerebral palsy (CP) and typically developing control children (Con)

	CP (n = 28)	Con (n = 18)	d	*p*
Age (years)	9.2 ± 1.9	9.0 ± 2.4	*0.097*	*0.937*
Sex (male/female)	18/10	13/5	—	—
Height (m)	1.34 ± 0.13	1.36 ± 0.15	*0.169*	*0.437*
Height (%)	47 ± 35	67 ± 22	*0.634*	*0.110*
Body mass (kg)	31.6 ± 7.6	32.2 ± 10.7	*0.067*	*0.826*
Body mass (%)	51 ± 33	60 ± 27	*0.198*	*0.597*
BMI	17.4 ± 3.0	16.9 ± 2.5	*0.223*	*0.605*
BMI (%)	54 ± 33	53 ± 27	*0.021*	*0.955*
Arm dominance (left/right)	15/13	0/18	—	—
CP diagnosis (unilateral/bilateral)	19/9	—	—	—
GMFCS level (I/II)	25/3	—	—	—
MACS level (I/II)	3/25	—	—	—
Mirror movement (present/absent)	14/14	—	—	—
Tested hand for individuation task	6/16/6	18/0/0	—	—
(preferred only/non-preferred only/both hands)				
Hypertonia Assessment Tool tested arm	20^a^/6^b^/4^c^/4^d^	—	—	—

Data are presented as mean ± SD. BMI, Body mass index; GMFCS, Gross Motor Function Classification System; MACS, Manual Ability Classification System; % for height, body mass, and BMI reflect percentiles relative to age- and sex-based norms. ^a^normal tone, ^b^spasticity, ^c^dystonia, ^d^mixed tone (both spasticity and dystonia present).

**Table 2:** Partial Pearson correlation coefficients (age-corrected) between overall Individuation Indexes and clinical assessment outcomes in children with cerebral palsy (CP) and typically developing control children (Con)

CP Non-preferred arm		CP Preferred arm	Con Preferred arm
	(n = 17)	(n = 11)	(n = 13)
	Individuation Index		
${\text{FDT score}}_{\text{normalized}}$	*r* = 0.15; *p* = 0.577	*r* = 0.55; *p* = 0.100	*r* = 0.11; *p* = 0.742
${\text{BBT score}}_{\text{normalized}}$	*r* = −0.10; *p* = 0.719	*r* = 0.56; *p* = 0.095	*r* = −0.25; *p* = 0.433
${\text{PPT score}}_{\text{normalized}}$	*r* = 0.03; *p* = 0.921	*r* = 0.62; *p* = 0.057	*r* = −0.52; *p* = 0.083

**r:** Partial Pearson correlation coefficient controlling for age.

## Abbreviations

BBT: Box and Blocks Test
BMI: Body mass index
Con: Typically developing control children
CP: Cerebral palsy
CST: Corticospinal tract
FDT: Functional Dexterity Test
GMFCS: Gross Motor Function Classification System
HAND: Hand Articulation Neuro-training Device
LMM: Linear mixed-effect model
MACS: Manual Ability Classification System
meanDevP: Mean deviation from baseline fingertip forces
PPT: Purdue Pegboard Test
SMC: Selective motor control
